# Development of a VNIR/SWIR Multispectral Imaging System for Vegetation Monitoring with Unmanned Aerial Vehicles

**DOI:** 10.3390/s19245507

**Published:** 2019-12-13

**Authors:** Alexander Jenal, Georg Bareth, Andreas Bolten, Caspar Kneer, Immanuel Weber, Jens Bongartz

**Affiliations:** 1 Application Center for Machine Learning and Sensor Technology, University of Applied Science Koblenz, 53424 Remagen, Germany; kneer@hs-koblenz.de (C.K.); immanuel.weber@hs-koblenz.de (I.W.); bongartz@hs-koblenz.de (J.B.); 2 Institute of Geography, GIS & RS Group, University of Cologne, 50923 Cologne, Germany; g.bareth@uni-koeln.de (G.B.); andreas.bolten@uni-koeln.de (A.B.)

**Keywords:** short-wave infrared (SWIR), visible (VIS), near-infrared (NIR), remote sensing, unmanned aerial vehicle (UAV), multispectral, precision agriculture, phenotyping, forestry, vegetation

## Abstract

Short-wave infrared (SWIR) imaging systems with unmanned aerial vehicles (UAVs) are rarely used for remote sensing applications, like for vegetation monitoring. The reasons are that in the past, sensor systems covering the SWIR range were too expensive, too heavy, or not performing well enough, as, in contrast, it is the case in the visible and near-infrared range (VNIR). Therefore, our main objective is the development of a novel modular two-channel multispectral imaging system with a broad spectral sensitivity from the visible to the short-wave infrared spectrum (approx. 400 nm to 1700 nm) that is compact, lightweight and energy-efficient enough for UAV-based remote sensing applications. Various established vegetation indices (VIs) for mapping vegetation traits can then be set up by selecting any suitable filter combination. The study describes the selection of the individual components, starting with suitable camera modules, the optical as well as the control and storage parts. Special bandpass filters are used to select the desired wavelengths to be captured. A unique flange system has been developed, which also allows the filters to be interchanged quickly in order to adapt the system to a new application in a short time. The characterization of the system was performed in the laboratory with an integrating sphere and a climatic chamber. Finally, the integration of the novel modular VNIR/SWIR imaging system into a UAV and a subsequent first outdoor test flight, in which the functionality was tested, are described.

## 1. Introduction

Multi- and hyperspectral data acquisition with unmanned aerial vehicles (UAVs), also known as unmanned aerial systems (UASs), remotely piloted aircraft systems (RPAS), or drones has been of significant research interest in vegetation remote sensing applications during the last decade [1,2,3,4,5]. Operating systems with take-off weights below 10 kg makes them very flexible for spatio-temporal data acquisition at high to ultra-high spatial resolution. Hence, monitoring and considering phenological change patterns in a new and more appropriate way is possible. For example, water, nutrients, or other stresses can be investigated and differentiated in more detail [6,7,8,9,10,11,12].

However, most operational multi- or hyperspectral pushbroom or snapshot sensors capture wavelengths from approx. 350 to 1000 nm, in the so-called visible to near-infrared (VNIR) domain [13]. Sensors also covering the short-wave infrared (SWIR) are rarely available for UAV-based applications. To the authors’ knowledge, the only exceptions are the SWIR sensor introduced by Honkavaara et al. [7] and Tuominen et al. [14], which is based on a tunable Fabry-Pérot interferometer (FPI) and pushbroom sensors as described in [15]. In this contribution, we follow the VNIR (400 to 1000 nm) and SWIR (1000 to 2500 nm) definitions by Stark et al. [16], which are mainly based on the spectral responses of the available image sensors (silicon and indium gallium arsenide—InGaAs, respectively). The advantage of frame sensors, in general, is that the captured images can also be used to create 3D spectral data and to correct for bidirectional reflectance distribution properties in images [1,17]. The combined analysis of structural and spectral data also seems to result in more robust estimators for vegetation traits [14,18,19,20,21,22,23].

The SWIR domain provides for remote sensing applications additional significant spectral absorption features, e.g., for geology [24,25] as well as for vegetation trait retrievals [26] using vegetation indices (VIs) like the Normalized Ratio Index (NRI) introduced by Thenkabail et al. [27]. Koppe et al. [28,29] showed that the two-band NRI using all possible band combinations in the VNIR/SWIR domain outperformed established VIs like Normalized Difference Vegetation Index (NDVI) for biomass monitoring of winter wheat by using a combination of NIR/SWIR wavelengths (874 nm and 1225 nm). Similar results are described by Gnyp et al. [30], who introduced a four-band vegetation index, the GnyLi, for winter wheat biomass monitoring, which uses wavelengths of NIR/SWIR absorption characteristics (900, 955, 1055 and 1220 nm). Similar results for the GnyLi were reported for barley by Bendig et al. [18] and Tilly et al. [31]. Besides biomass, nitrogen uptake and nitrogen concentration are of crucial importance in vegetation trait monitoring. Camino et al. [15] investigated the potential of SWIR bands to monitor nitrogen concentration in winter wheat. The authors conclude that established VIs like the Transformed Chlorophyll Absorption Reflectance Index (TCARI), the Modified Chlorophyll Absorption in Reflectance Index (MCARI), or the Optimized Soil Adjusted Vegetation Index (OSAVI) perform significantly better when using a SWIR version of these VIs using 1510 nm, i.e., the TCARI_1510_, the MCARI_1510_, and the OSAVI_1510_. Tilly and Bareth [20] investigated the GnyLi for nitrogen and described very promising results. For the analysis of the water status of vegetation, for example, Gao [32] introduced the Normalized Difference Water Index (NDWI). The NDWI utilizes spectral properties at 860 nm and 1240 nm in an NDVI-like equation mentioned in Haboudane et al. [33]. Similarly, the Moisture Stress Index (MSI) performs as a simple ration index using a SWIR and NIR wavelength. Further, Serrano et al. [34] introduced the Normalized Difference Nitrogen Index (NDNI) using 1510 nm and 1680 nm and the Normalized Difference Lignin Index (NDLI) using 1680 nm and 1754 nm. A comprehensive review of hyperspectral vegetation indices is given lately by Roberts et al. [35]. Summarizing the published studies using the VNIR/SWIR domain for determining vegetation traits, the additional usage of SWIR bands improves the detection of (i) biomass, (ii) nitrogen, and (iii) stress factors like water stress.

The research demand to evaluate and validate the true potential of the VNIR/SWIR domain for vegetation monitoring is very high due to its potential to derive more robust spectral estimators for vegetation traits also in combination with structural data analysis [15,20,23]. Therefore, a VNIR/SWIR imaging system for UAVs is desired, which enables data acquisition in ultra-high spatial resolution and for certain phenological stages resulting in a daily or weekly temporal resolution. The VNIR/SWIR imaging system should also be able to capture selectable spectral bands for the purposes mentioned above, like the NDWI or GnyLi (e.g., 860, 900, 970, 1050 and 1240 nm).

This paper presents the development of a novel and first VNIR/SWIR multi-camera 2D imaging system for UAVs, which combines two VNIR/SWIR sensitive cameras that are capable of selecting two wavelength bands from 400 nm to 1700 nm in parallel, with a custom-designed filter integration solution for fast interchanging filter setups.

The system was designed to be modular and expandable for use with a variety of airborne carrier platforms, especially for small to medium off-the-shelf UAVs (sUAVs) with a potential payload of 1.5 kg or more. In addition, the backend of the system offers additional capabilities to integrate more image sensors in the future to simultaneously acquire up to four wavelength bands. Since the VIS/NIR range is already covered by a large number of commercially available multispectral multi-camera systems [13] (e.g., MicaSense [36], MAIA [37], Parrot Sequoia [38], and Tetracam [39]), this newly developed VNIR/SWIR imaging system is specially designed for wavelength detection in the SWIR spectral range. Especially already developed and validated VIs such as NDWI, NRI, or GnyLi can be examined by this system with a higher spatio-temporal resolution. Therefore, four wavelengths relevant for the NDWI, the NRI, and GynLi (910, 980, 1100, and 1200 nm) were selected and used for the basic setup and tests. In principle, however, any filter combination from the VNIR/SWIR spectral range of 400–1700 nm is possible for UAV-based remote sensing applications.

**Figure 1:**
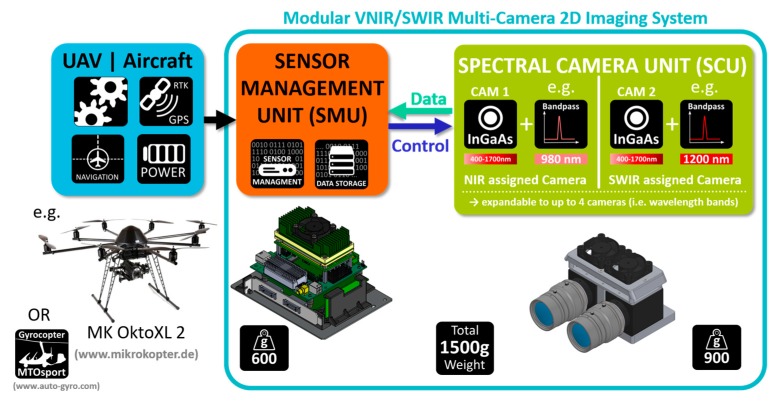
Schematic overview of the complete UAV-based VNIR/SWIR imaging system.

Section 2 describes the hardware design and implementation process of the overall VNIR/SWIR imaging system (Figure 1) in detail and begins with an overview of the spectral camera unit (SCU, Section 2.1) that is further divided into the single optical components which are the selected VNIR/SWIR camera sensors (Section 2.1.1) and lenses (Section 2.1.2) and the assembly layout for the optical filter placement, namely the internal filter assembly (Section 2.1.3). Section 2.1.4 presents an overview of the mechanical structure of the SCU and considerations for a thermally optimized design. The second part of the camera system, the sensor management unit (SMU), is described in Section 2.2. There the main components are delineated in detail. These include the selected frame grabber (Section 2.2.1), the computer unit (Section 2.2.2), and a custom-designed printed circuit board (Section 2.2.3). Section 3 gives an insight into the methodology of the preliminary characterization of the camera system with regard to the thermal and optical properties. Section 4 presents the obtained test results of the individual components and the overall system outlined in Section 2, as well as the characterization results of the parameters described in Section 3.

## 2. Hardware Design

The system setup was designed modularly to improve usability for UAV-based image acquisition. Therefore, the system is divided into two modules (see Figure 1). The first one is the spectral camera unit (SCU), which consists of two InGaAs-based image sensors in the form of two machine vision cameras. The second part is the sensor management unit (SMU) that combines all components that are necessary to operate the system altogether. Computer-aided design (CAD) software was used to implement and adapt the system with specially developed parts. For rapid prototyping and manufacturing of the designed parts, an in-house industrial 3D production system was available.

### 2.1. Spectral Camera Unit (SCU)

#### 2.1.1. VNIR/SWIR Image Sensor

**Figure 2:**
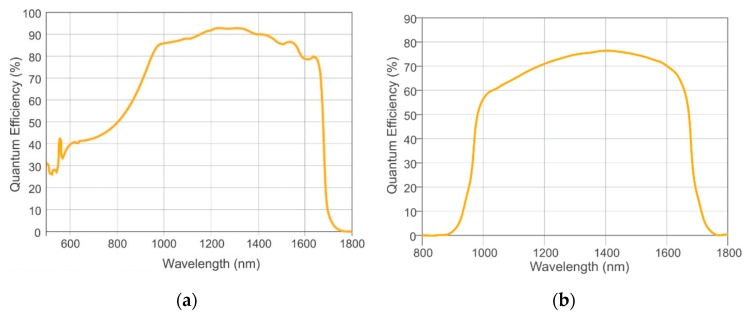
(**a**) Quantum efficiency of the used VIS-NIR enhanced InGaAs Sensor (https://www.raptorphotonics.com/products/owl-640-tecless-vis-swir-ingaas/); (**b**) Quantum Efficiency of a standard InGaAs Sensor (https://www.raptorphotonics.com/products/owl-640-swir/).

SWIR sensitive camera modules were comparatively heavy in weight and complex in the past. Also, the high costs for a camera and the necessary accessories (lenses, filters) were exclusion criteria for many applications. However, due to the progress achieved in recent years both in sensor technology and in microelectronics [40], and the increased production volume for industrial requirements, new camera modules that no longer have these disadvantages are now available. So-called SWaP cameras represent the latest trend in this optimization process, which focuses on the reduction of “size, weight and power”. The Spectral Camera Unit (SCU) consists of two such OWL 640 Mini VIS-SWIR SWaP camera modules manufactured by Raptor Photonics [41]. The core component of each module is an indium gallium arsenide (InGaAs) photodiode array (PDA) with 640 × 512 pixels (quasi VGA) and 15 µm pixel pitch. In contrast to the common photosensitivity of InGaAs focal plane arrays (FPA), with wavelengths between 900 nm and 1700 nm, this back-illuminated sensor has an extended sensitivity range down to approx. 400 nm (Figure 2) [42].

Due to a further etching step during production, the indium phosphor (InP) passivation layer on top of the InGaAs layer is thinned out, so that also visible photons can pass through [42,43,44]. Due to the small bandgap of InGaAs, electrons can pass more easily from the valence band into the conduction band by thermal excitation, which leads to an increased intrinsic dark current compared to their silicon counterparts [42,45]. According to the SWaP approach, however, the cameras do not have an integrated thermoelectric cooler (TEC) for temperature stabilization of the sensor. This produces a higher dark noise level. However, this increased noise floor is acceptable as the developed system is not intended for night vision applications but for bright daylight conditions, and therefore the shot noise of the photons determines both the dynamic range (DR) and the signal-to-noise ratio (SNR). The missing TEC, i.e., Peltier element, results in reduced power consumption of less than 2.5 W as well as a noticeably lower unit price. With a size of 60 mm × 42 mm × 42 mm and a weight of 170 g of a single camera module, it is compact enough to fit at least two units in an UAV-gimbal.

Both cameras output 14-bit image data via the CameraLink protocol (in base configuration). Therefore, the cameras cannot directly be connected to a computer like a USB3 Vision or a GigE Vision device. A frame grabber has to join up in circuit to transfer data from the cameras to a host computer. The frame grabber is integrated into the sensor management unit (SMU) and is described below (Section 2.2). The SCU is therefore connected via two high-flexible CameraLink cables to the frame grabber unit. The sensor has a global shutter, which is inevitable for synchronized readout and artifact-free images [13] during flight. Due to a universal clamping device, the camera body offers the advantage of creating even non-standard lens mounts via adaptable threaded flanges. The camera is supplied with a c-mount flange as standard. Since the two cameras are not completely identical in their properties and for the stringent allocation of the wavelengths during the measurement, each of the two cameras is allocated to a spectral range and named after it for better differentiation. The NIR filter camera (NFC) is primarily only used with NIR filters (up to 1000 nm) and the SWIR filter camera (SFC) only with filters from the SWIR spectral range (1000–1700 nm). In principle, the lower wavelength is always assigned to the NFC and the higher to the SFC.

#### 2.1.2. VNIR/SWIR Camera Lens

Simultaneously with the selection of a suitable InGaAs camera, a suitable lens was also selected [46]. For each channel, the incident light passes through a Kowa LM12HC-SW lens (www.kowa-lenses.com) with a nominal wide-angle fixed focal length of 12.5 mm and a minimum f-stop of f/1.4. The lens is designed for 1” sensors, and the spectral transmission is optimized for the infrared range from 800 nm to 2000 nm. This wavelength range is sufficient for later use in the NIR and SWIR spectral range. However, if wavelengths below 800 nm become interesting, either a pure VIS or directly a VNIR/SWIR capable lens must be used. The wide-angle focal length was chosen to best fit the required ground sampling distance (GSD) of less than 0.1 m with regard to the flying altitudes of different aircraft like UAVs as well as microlights, e.g., gyrocopters. This helps, even for low flying altitudes, to minimize the amount of image data while sufficient ground coverage and image overlap for Structure from Motion (SfM) and Multi-View Stereopsis (MVS) algorithms are still guaranteed. Flight altitudes of 30 m above ground level (AGL) are envisaged for planned UAV applications. In combination with the sensor parameters, the various flight planning parameters of the system can be derived [47,48]. The angle of view (AoV) equals 27.51° in the vertical, 42.01° in the horizontal, and 48.79° in the diagonal. The respective field of view (FoV) at a working distance of 30 m results in 23.04 m horizontally, 14.69 m vertically, and 27.32 m diagonally with a ground sampling distance (GSD) of about 0.04 m.

#### 2.1.3. Filter Assembly Layout for Spectral Band Selection

**Figure 3:**
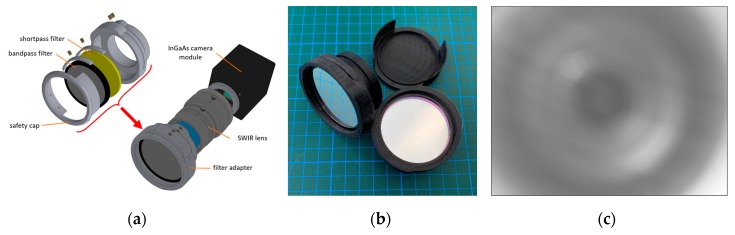
(**a**) Exploded view of the developed adapter for the filter front-assembly; (**b**) Final version of the plug-in adapter with inserted filter elements and protective cap; (**c**) Flat-field image with ring-like mirroring artifacts. This design approach was discarded because of these ambiguous artifacts.

With the prime lens attached to the camera body, the imaging system is only capable of detecting the cumulated incoming light in the range of the spectral transmission of the lens as well as of the spectral response of the sensor. In order to select a specific and application-oriented spectral band out of this broadband spectrum, usually a bandpass filter with the best fitting central wavelength (CWL) and narrow bandwidth (BW) has to be integrated into the optical path. In general, there are only two possible layouts to mount the filter in the optical path, as described by Wang et al. [49]. One solution is to mount the filter in front of the lens (front-assembly, Figure 3). In order to test this approach, special snap-on adapters for front-mounting the filter discs were designed and manufactured. Although this filter front-assembly is easier to implement and more practical to use, the optical characterization tests have shown that this configuration produces ring-like image artifacts. These are probably generated by mirror effects on the individual anti-reflective coatings of the filters and the individual lens. As both the exact origin and the quantification of these artifacts could not be clearly determined, this approach was not further pursued and was discarded during the development process.

The second possible layout is the placement of the filter between the lens and the sensor [49]. If the given form factor of the camera body allows it at all, this layout is more complex to realize. Also, for this solution, it is usually only possible to use existing off-the-shelf filter elements, which in turn means a limitation in both diameter and spectral properties.

Moreover, there are also two options for this internal approach. Firstly, a single interference filter mounted in a threaded ring can be screwed into the C-Mount thread. However, this has the disadvantage that the blocking range of these single filter elements is not sufficient to cover the complete sensitivity of the VIS-NIR enhanced InGaAs sensor. This means that ordinary bandpass filters become transparent again to higher wavelengths above a specific cut-off wavelength, and thus light from unwanted spectral bands can reach the sensor. Fortunately, there are also hybrid filter elements with an ultra-wide blocking range available [50]. These hybrid filters consist of different filter elements, like interference and absorption filters, as well as a Fabry-Perot cavity, which are perfectly matched to each other. Additionally, the FWHM is reduced to 10 nm for the two SWIR filters. A drawback of this filter structure is that the materials which increase the blocking range also decrease the overall transmission in the passband [50].

Table 1 lists the detailed specifications of the selected filter elements so far used in the spectral camera system. The central wavelengths of the bandpass filters were selected for later use in the NIR/SWIR domain.

**Figure 4:**
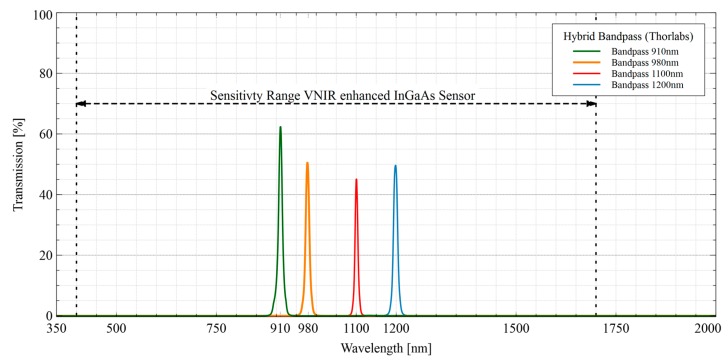
Transmission data of the used hybrid bandpass filters provided by the manufacturer (www.thorlabs.com).

Figure 4 shows the transmission data of the four used bandpass filters provided by the manufacturer (www.thorlabs.com). Compared to conventional hard-coated interference filters with typical transmissions of over 90%, the reduced transmissions can be seen in the passband of the individual hybrid filter elements. On the other hand, these filters have an ultra-wide blocking range, which covers the entire spectral sensitivity of the sensor.

**Figure 5:**
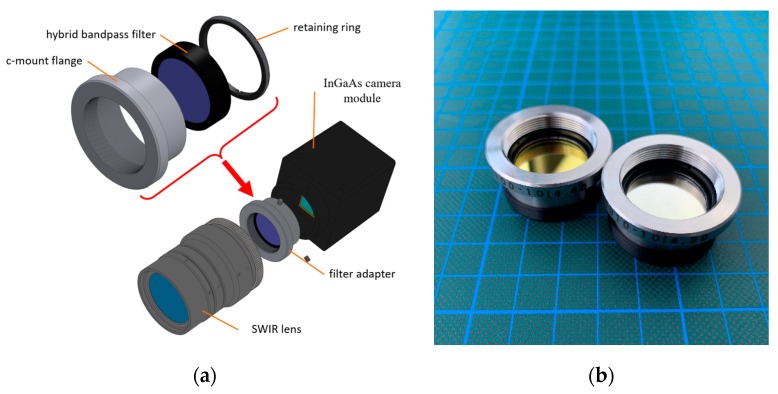
(**a**) Exploded view of the custom-designed c-mount flange for the inlay filter assembly between lens and sensor; (**b**) Set of two equipped c-mount flanges with hybrid filters inserted.

Moreover, the camera mount type, here c-mount, defines the flange focal distance of a lens. Every optical component introduced into this path changes the focus characteristics of the lens, which requires compensation by appropriate measures [49]. This can, for example, be achieved with an additional tube with focusing lens elements. In order to avoid these adjustments, a different solution was attempted, namely, to integrate the hybrid filters directly into the lens mount. As the lens mount of the used camera is replaceable, a custom c-mount flange could be designed (Figure 5). This component fulfills several tasks. The lens is mounted to it, the distance for adjusting the focus is compensated, and the individual hybrid bandpass filter element is placed as desired and is fixed with a retainer ring. A further advantage is the more parallel beam path in comparison to the wide-angle entrance area of the lens and the resulting smaller angle of incidence (AOI) on the filter. This reduces the possible blue-shift introduced by the wide-angle focal length of the lens [16]. A further advantage is that these hybrid filters are also significantly cheaper than the large filter discs (50 mm), necessary for the front-assembly, due to their smaller diameter (25.4 mm).

#### 2.1.4. Mechanical and Thermal Design

**Figure 6:**
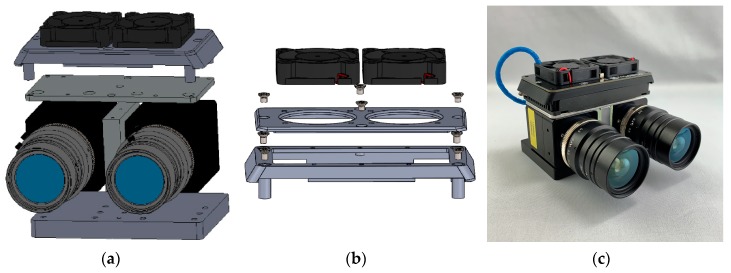
(**a**) Exploded view of the mechanical construction of the Spectral Camera Unit with an optional fan adapter applied; (**b**) Exploded view of the CAD model of the fan adapter of the SCU for thermal stabilization; (**c**) Current state of development of the SCU.

The exact parallel alignment of the two camera modules with each other is achieved by a specially designed and 3D printed mounting plate (see Figure 6a,c). This mounting plate also allows the two cameras to be easily mounted and aligned into a gimbal or on a tripod. As mentioned in Section 2.1.1, the cameras have no integrated temperature stabilization circuit (TEC). Unlike other camera models from different manufacturers, the Owl 640 mini can be operated without a further external cooling system as long as the operating case temperature of +55 °C is not exceeded. If the camera is operated above this limit, the ohmic heat can cause permanent damage to the sensor and the electronics.

Moreover, sensor degradation is reduced with sensor temperatures below +40 °C, as well as the signal-to-noise ratio. Therefore, the two camera modules are thermally coupled and connected to a heatsink on top of the camera bodies (see Figure 6b). To dissipate the heat more effectively, a specialized heatsink attachment that can be equipped with one or two fans was designed and 3D printed. Therefore, the system can be adapted to higher ambient temperatures. Attempts to cool the sensor with an external TEC were not successful. On the one hand, the operation of the TEC requires much energy and a correspondingly large and bulky heat sink. On the other hand, the cooling effect from outside the housing is far less effective than a TEC directly integrated into the image sensor circuit.

### 2.2. Sensor Management Unit (SMU)

**Figure 7:**
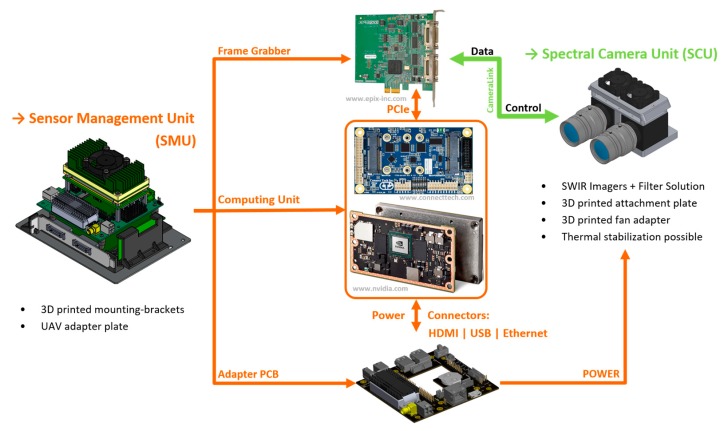
Detailed schematic view of the three main components of the Sensor Management Unit (SMU), and the interconnection of the single parts and the Spectral Camera Unit (SCU).

**Figure 8:**
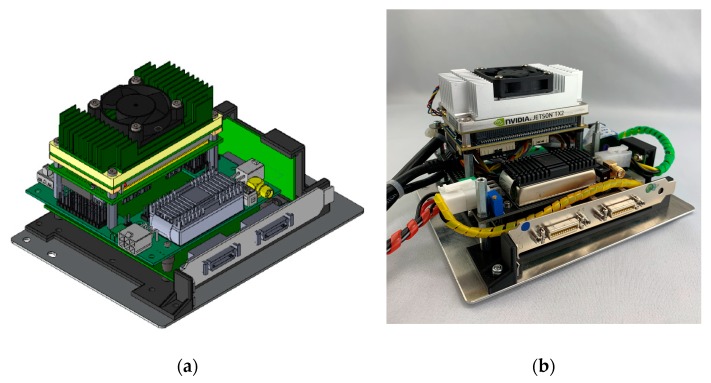
(**a**) CAD side view of the assembled sensor management unit; (**b**) The current state of development of the SMU.

The SMU (Figure 7) is layered from several individual components to form a compact unit (Figure 8), which can then be integrated as part of the overall imaging system, e.g., into a UAV. The unit consists of three main components:

- Frame grabber
- Adapter PCB
- Computing unit

and fulfills the following tasks:

- connecting the SCU to the computing unit via a frame grabber,
- providing regulated power with safety features to the overall system,
- controlling the two cameras of the SCU,
- storing the image data from the SCU,
- providing connections for additional hardware and future extensions like GNSS or IMU,
- providing enough computing power for future direct onboard image processing and machine learning tasks,
- providing further interfaces for additional cameras.

The individual components are described in detail below.

#### 2.2.1. Frame Grabber

A dual CameraLink frame grabber card (base configuration, Epix Inc., www.epix-inc.com) serves as a bridge between the single cameras of the SCU and the computing unit. The frame grabber card is initially intended for installation in a desktop PC case and is usually connected to the mainboard via a PCI Express x1 Expansion Slot. Therefore, a mounting bracket adapted to the card was designed, and 3D printed to securely attach it to an adapter plate for the UAV integration. Epix offers a software (XCAP) to control the cameras and obtain image data via the frame grabber. Furthermore, the company provides its own “C” programming libraries (XCLIB) to access the frame grabber from self-written programs or graphical user interface (GUI) applications. These libraries are available for Windows as well as for various Linux operating systems. A GUI application has been implemented with the Epix application programming interface (API) for camera control and storing the acquired image data.

#### 2.2.2. Computing Unit (CU)

**Figure 9:**
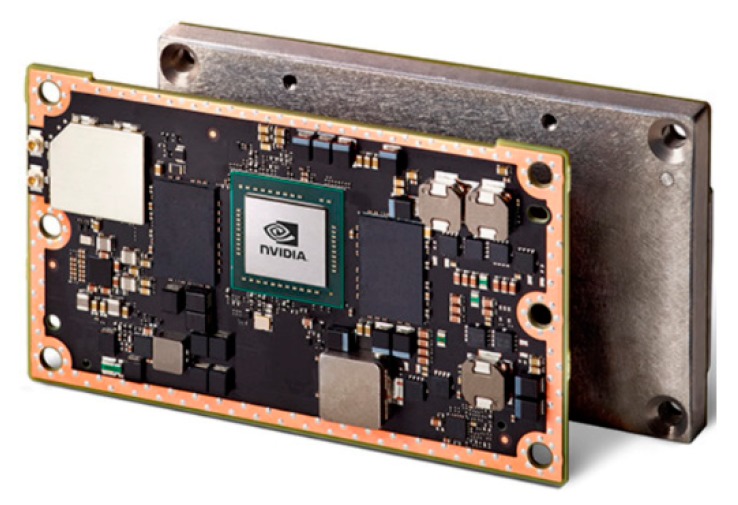
Nvidia Jetson TX2 system on module (SoM) and Thermal transfer plate (TTP) (courtesy of NVIDIA).

The centerpiece of the computing unit is the Nvidia Jetson TX2 embedded system-on-module (SoM, www.nvidia.com, Figure 9). This little brick-shaped module is slightly larger than a credit card (50 × 87 mm) and weighs 85 g, including a so-called thermal transfer plate (TTP), but it combines all active processing components of a powerful computer

For additional heat dumping in high-power mode, there is a fan-powered heatsink attached to the TTP. This small computer module runs with Ubuntu 16.04 LTS. The initial task for the TX2 is to run a specifically programmed software that can both control the cameras and read and store the image data from the cameras.

**Figure 10:**
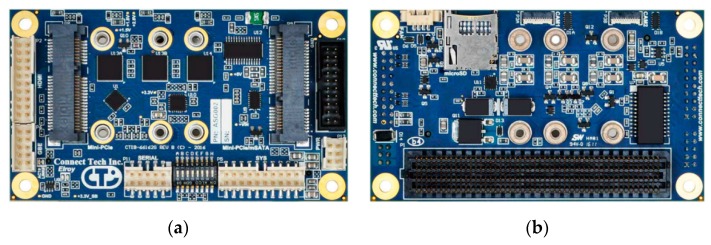
(**a**) Front view of the top side of the Connect Tech Elroy Carrier; (**b**) Front view of the bottom side of the Connect Tech Elroy Carrier (courtesy of Connect Tech Inc.).

Since the TX2 is just a bare SoM, all signals, power rails, and the standard hardware interfaces are only accessible via a 400-pin connector. All these interconnections have to be broken out through different application-specific carrier boards provided by third-party companies. Therefore, the Elroy Carrier (EC) from Connect Tech (http://connecttech.com, Figure 10) was selected for the intended use of the TX2. In order to use the TX2, it was configured with the appropriate board support packages (BSP) for the EC and then mounted on the carrier board for the implementation in the SMU. The Elroy Carrier matches the TX2 form-factor and provides most of the physical interfaces of the TX2. These are necessary to connect additional hardware such as SSD memory or the frame grabber card.

The mini-PCIe slot of the carrier can connect the Epix frame grabber via a mini-PCIe to PCIe adapter. An additional mSata slot on the EC extends the internal 30 GB memory of the TX2 with a half-sized 128 GB mSata SSD module. The remaining connections are provided by several pin-header ports that can be interconnected with prefabricated cable assemblies from Connect Tech.

#### 2.2.3. Adapter Circuit

**Figure 11:**
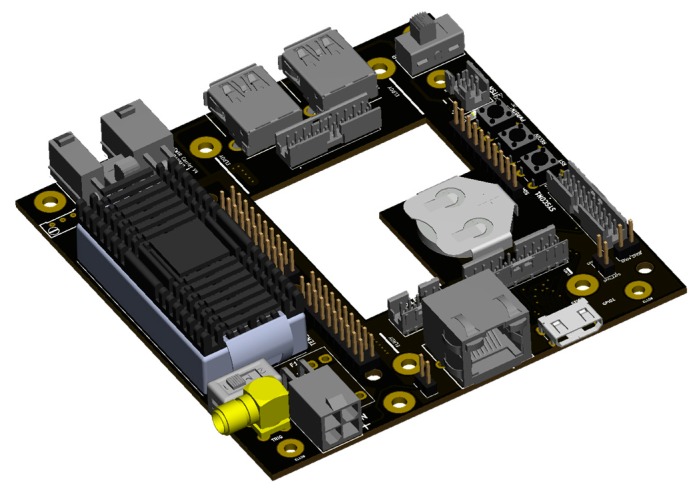
CAD View of the custom-designed and assembled adapter PCB.

A printed circuit board (PCB, Figure 11) was developed to integrate the Jetson TX2 electronically and mechanically into the SMU. The latest revision (V4) fulfills three main tasks:

(1) Regulated power supply

Unlike other machine vision camera modules, the OWL 640 mini has only a very close tolerance regarding the supply voltage (12 V DC ±10%). Therefore, an isolated DC/DC converter was integrated into the PCB. This electronic part provides a constant voltage (12 V, 30 W max) for the overall system form a wide input voltage range (9 V to 36 V) and includes critical safety features like over-voltage protection, under-voltage lockout, over-temperature, and short-circuit protection.

(2) Mechanical Connection layer

The form factor of the Elroy carrier board allows direct electrical and mechanical mounting to the TX2 and forming the computing unit (CU). However, to establish a suitable mechanical connection of the CU to the frame grabber, an additional connection layer is required. Here the board translates the drilling pattern of the frame grabber to that of the TX2.

(3) Electronic connection and expansion layer

As described above, the Elroy Carrier provides the TX2 with a variety of hardware interfaces to connect the computer module to other hardware. In some cases, however, these components must be physically placed outside the Elroy Carrier and then connected via the appropriate cable connectors. Here the board is mainly used to accommodate pushbuttons, status LEDs, and interfaces such as USB, HDMI and Ethernet, which would otherwise have to be connected via bulky cables. The trigger inputs of the camera modules can be connected to the adapter board via an SMA connector with a coaxial cable. The SMA connector is connected via the PCB to one of the two GPIO inputs of the TX2. Most of the CU connectors are connected via pin-header sockets.

## 3. Methods for Preliminary Camera System Characterization and Tests

### 3.1. Thermal Management and Dark Noise

**Figure 12:**
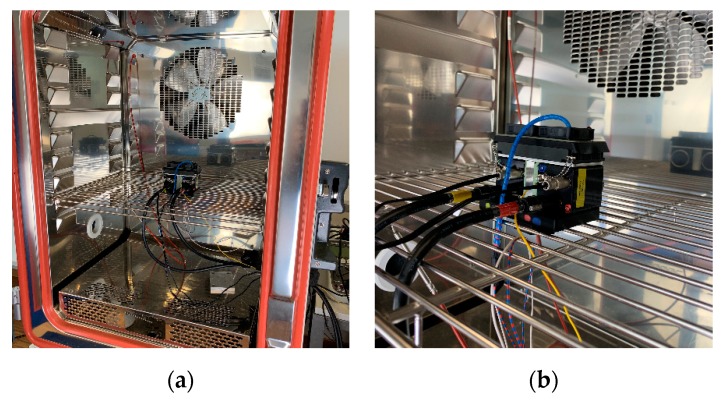
Measurement setup for thermal camera performance in a climatic chamber (Weiss WK11 180) at the test laboratory at the RheinAhrCampus of the University of Applied Science Koblenz: (**a**) Placement of the SCU in the climatic chamber; (**b**) Detailed view of the SCU into the climatic chamber.

Self-heating is a determining factor for the magnitude of the various temperature-dependent sources of interference in image sensors, which in turn degrade the image quality [45,51,52]. In principle, it is possible to read the temperature data of both the image sensor (CCD) and the electronics (PCB) of the OWL 640 mini camera module. This makes it possible to check the effectiveness of the implemented external thermal stabilization solution (see Section 2.1.4) and to use the thermal information for later calibration procedures. For the thermal examinations, the SCU was inserted in a climatic chamber (Weiss WK11-180, www.weiss-technik.com, Figure 12). In order to check the thermal design considerations described in Section 2.1.4, the two cameras in the SCU were operated with the external fans switched off. The resulting warm-up time of the image sensor and electronics was recorded at regular intervals with a constant ambient temperature in the climatic chamber and without a stabilized ambient temperature outside the chamber. Also, the ambient, as well as the enclosure temperatures, were logged. After a constant temperature level had been set inside the two cameras, the two external fans were activated to accelerate heat dissipation. The resulting temperature change was again recorded until a new saturation point was reached.

As an ideal image sensor would not generate any signal in the absence of light and would only convert the light quanta hitting the single pixels into digital values when illuminated, real sensors, however, have undesirable effects due to various manufacturing inadequacies and material properties, which occur as disturbing effects in the image. In order to perform the most accurate and repeatable measurement possible, these imperfections must be determined at the sensor as well as at the pixel level and have to be reduced or eliminated by corrective measures. These effects can be measured in the absolute absence of light and are subsumed under the term dark signal [53]. Subsequently, in order to determine the resulting dark signal of the two InGaAs sensors a more complex series of measurements were carried out in which several constant ambient temperatures were set in the climatic chamber (5 °C, 10 °C, 15 °C, 20 °C, 25 °C, and 30 °C). At each temperature point, a series of different exposure times were set, and 120 dark images were taken per exposure time for both sensors. Different statistical values were then calculated from these image data. These include the average dark signal and the fixed-pattern noise or dark-signal non-uniformity (DSNU) [54,55,56]. The test procedure and the calculation of the statistic values were based on the specifications of the EMVA1288 standard [57] and Mansouri et al. [58].

### 3.2. Evaluating the Transmission of the Optical System

**Figure 13:**
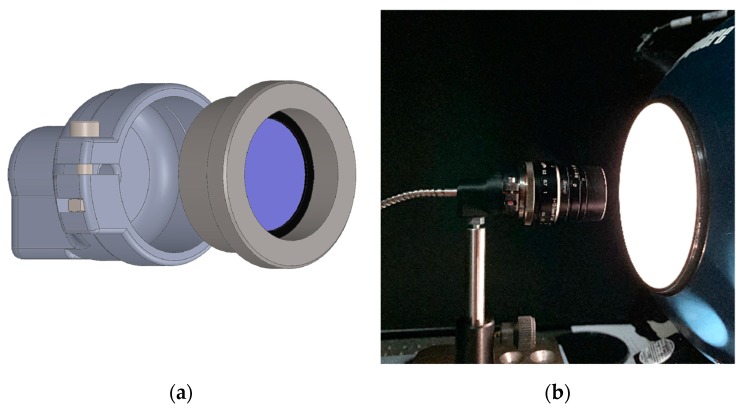
(**a**) Exploded view of the custom-designed fiber mounting adapter; (**b**) Measurement setup for transmission tests of the hybrid filter solutions (see also Figure 5) with an integrating sphere and an ASD FieldSpec 4 Wide-Res field spectroradiometer at the spectral laboratory at the IBG-2 at the research center Forschungszentrum Jülich, Germany.

The complete optics of the two camera channels consisting of the individual lens (2.1.2) and the particular filter combination and layout (2.1.3) were spectrally examined for compliance with the data provided by the manufacturers (see Figure 4). The spectral measurements were performed with an ASD FieldSpec 4 Wide-Res field spectroradiometer (https://www.malvernpanalytical.com/), and an integrating sphere (Labsphere CSTM-USS-1200C-SL, https://www.labsphere.com/) placed in the spectral laboratory at the Plant Sciences (IBG-2) at the research center Forschungszentrum (FZ) Jülich GmbH, Germany (www.fz-juelich.de/ibg/ibg-2). Figure 13 shows the measurement setup for the internal assembly layout. For testing purposes, the fully equipped filter flanges were each mounted in a specially designed holding device (Figure 13a). This is similar to the filter flange mount on the camera housing and places the front surface of the spectrometer fiber in the focal plane of the lens. This setup was then placed in front of the opening of the integrating sphere. The settings of the lens (aperture, focus) were kept constant for all measurements and are based on the settings for later use in the field.

### 3.3. Flat-Field Measurements

**Figure 14:**
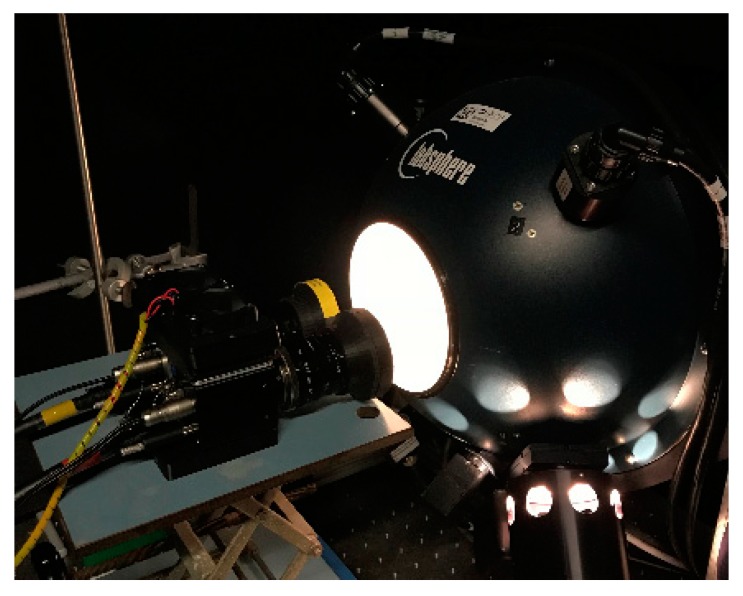
Measurement setup for the Spectral Camera Unit with the integrating sphere: for evaluation of the two different filter assemblies. The figure shows the test setup for the snap-on adapters of the front-assembly, which were no longer used due to artifacts.

In a further step, the imaging properties of the entire optical system (SCU), including the sensor, were examined. Together with the filter adapters, the lenses were flange-mounted to the cameras, and the resulting imaging system was evaluated with the uniformly illuminated integrating sphere. Figure 14 shows the measurement setup with the front-assembly configuration as the device under test (DUT).

### 3.4. UAV Integration of the VNIR/SWIR Imaging System

The complete VNIR/SWIR imaging system was test-fitted to a Mikrokopter MK 6S12 (www.mikrokopter.de). This multi-rotor UAV has a maximum payload of around 2.5 kg. Depending on the payload and the batteries, the flight time varies between 15 and 30 min. Altitude, airspeed, and flight paths are controlled by a GNSS, a pressure sensor, and pre-defined waypoints that can be configured via the MicroKopterTool. The same system was effectively used to implement prototype sensors on a UAV (Yara-N-Sensor, Bareth et al. [59]; Cubert Firefly, Aasen et al. [1], Aasen and Bolten [17]). In the present study, the UAV is equipped with two 5800 mAh, 129 WH LiPo-batteries, and an additional one-hour power supply for the imaging system. The take-off weight of the complete system is around 7.2 kg. The estimated flight time is around 15 min, depending on wind speed and altitude changes. For more details on the UAV, please refer to [1]. Popular large scale UAV like DJI Matrice 600 should reach a flight time around 30 min based on the flight time diagram on the manufacturer website (https://www.dji.com/matrice600).

**Figure 15:**
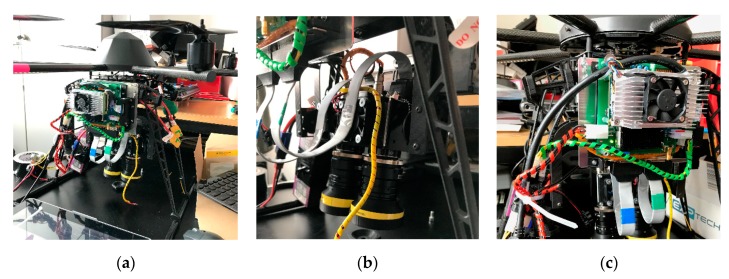
(**a**) Front view of all adapted system components (SCU and SMU); (**b**) Spectral camera unit mounted in the gimbal of the UAS; (**c**) Side view of the SMU adapted to the frame of the UAS.

The modularized two-part structure made it possible to connect the sensor managing unit (SMU) firmly to the frame of the UAV without having to change its entire structure. The spectral camera unit (SCU) was then mounted in the gimbal and connected to the SMU via flexible CameraLink ribbon cables. The gimbal uses two tilt servos to adjust pitch and roll and hold the system during the whole flight in a nadir position also outside of the balance point of the system. Figure 15 shows the individual parts mounted to the UAV.

## 4. Results of the Prototype Camera System Characterization and Tests

Of utmost importance for developing a sensor system for UAV-based applications is the evaluation of the sensor itself [58,60,61,62], the integration in a UAV platform, and the validation of the complete sensing system. Therefore, we present firstly evaluation results of the newly developed VNIR/SWIR imaging system and secondly first test data captured in operational UAV mode.

### 4.1. Thermal Management and Dark Signal

#### 4.1.1. Thermal Management

**Figure 16:**
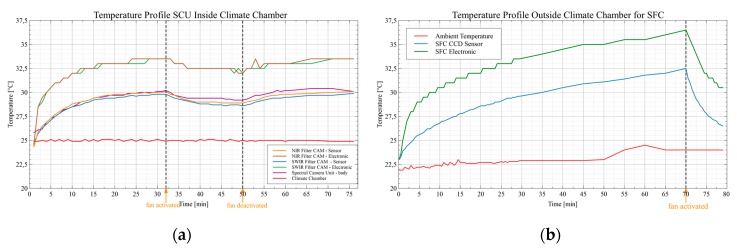
Influence of the temperature management on the interior temperature of the camera: (**a**) Temperature behavior of the NIR filter assigned camera (NFC) and the SWIR filter assigned camera (SFC) at 25 °C constant ambient temperature within a climatic chamber; (**b**) Temperature behavior of the SWIR filter camera (SFC) at an unregulated ambient temperature outside a climatic chamber.

As described in Section 3.1, temperature sensors can be read out at two points (sensor and control electronics) in each camera using special software. Figure 16a shows the temperature profiles for both camera modules in the climatic chamber at a set ambient temperature of 25 °C. It can be observed that the two modules have settled to a constant temperature after about half an hour. Activating the fan system after 32 min hardly reduces the internal temperature. This circumstance can be explained by the way the climatic chamber works, which circulates the air in the chamber through a fan in order to keep the temperature inside constant. This airflow also effectively removes heat from the SCU and thus virtually replaces the fan system. However, Figure 16b shows the temperature profile of the SWIR Filter assigned Camera (SFC) outside the climatic chamber. After switching on, the temperature slowly increases until the control electronics reaches a temperature of 36.5 °C, and the fan system is activated. After the activation of the fan system, a definite temperature drop of 6 °C is to be recognized in each case, although the ambient temperature increased by 3 °C during the measurement. Hence, the self-developed cooling system works as expected. The performance of the fans used in the design is adapted in such a way that a maximum cooling effect is guaranteed with the least possible influence on the overall system by electromagnetic interference (EMI) or vibrations. During the test phase, therefore, no influence of the fan system on the cameras with regard to EMI or motion artifacts due to vibrations in the image data was detected.

#### 4.1.2. Dark Signal

**Figure 17:**
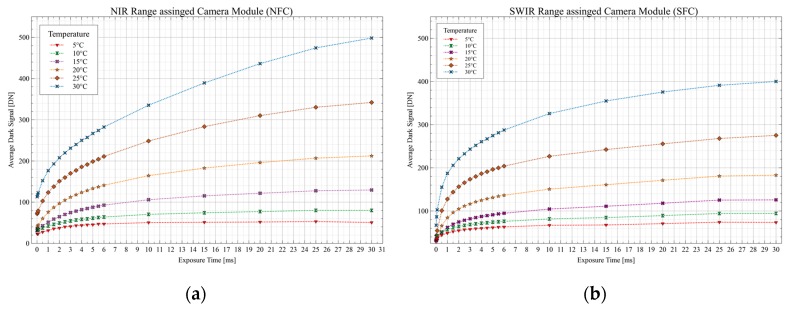
Climatic chamber measurements of the average dark signal for sensor characterization: (**a**) Average dark signal for the NIR range camera module; (**b**) average dark signal for the SWIR range camera module.

With the dark images taken at different constant temperatures with varying exposure times, the average dark signal can be determined [53]. For each exposure time, 120 dark images were recorded and then averaged. The resulting image is then averaged pixel-wise. The results for the temperature behavior of the two sensors (NFC and SFC) are displayed in Figure 17. The profile of the average dark signal can be determined as a function of the exposure time and contains a fixed offset and a thermal dependent component, the so-called dark current, which in the end is the average dark signal. Based on the theoretically 14-bit (16384 Digital Numbers—DNs) resolution of the sensor, even the peak value determined for the average dark signal (498 DN @30 °C|30 ms) is not the decisive factor and negligible when used in daylight remote sensing applications. Here, the light signal itself, i.e., the shot noise, is the dominant factor for determining the maximum signal-to-noise ratio (SNR_Peak_). This corresponds to the square root of the number of incoming photons or to the square root of the full well capacity of the pixels. Outdoor experiments revealed exposure times, as used in later flight scenarios, between 0.5 and 4 ms to be selected. This means that an exposure time of 30 ms is not used in later applications and that the average dark signal will be far below 500 DNs, at 30 °C and 30 ms for the NFC, in the range between 100 and 250 DN.

**Figure 18:**
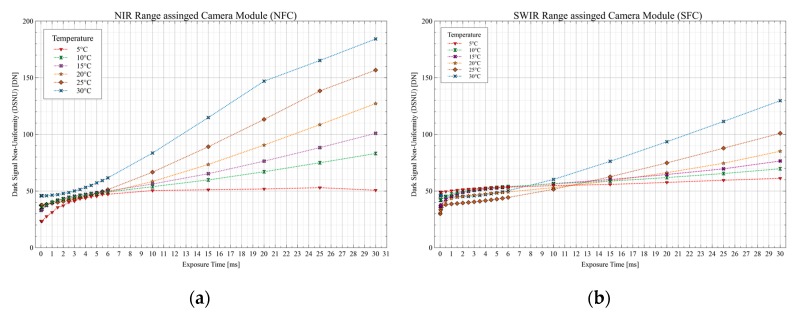
Fixed-pattern noise or dark signal non-uniformities (DSNU) as a function of various temperatures: (**a**) DSNU for the NIR range camera module; (**b**) DSNU for the SWIR range camera module.

Based on the average dark signal, the fixed-pattern noise (FPN), also called the dark signal non-uniformity (DSNU), which represents the variations of the dark signal between the individual pixels [54], can be calculated. This is the standard deviation of the pixel values for the averaged image of an exposure time at a specific set temperature. The DSNU is shown in Figure 18. As expected, the inter-pixel variation in the dark increases with increasing temperature and exposure time. However, also the FPN of 184 DN for the NFC sensor offers more than sufficient dynamic range for the 14-bit sensor at 30 °C and an exposure time of 30 ms, which is the upper range of the general operating limits.

### 4.2. Properties of the Optical System and Filter Layout

**Figure 19:**
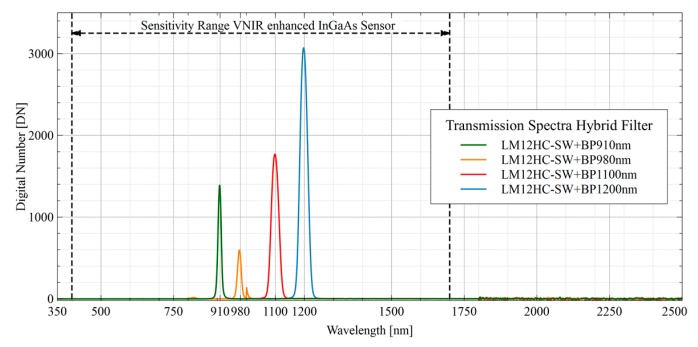
Recorded transmission data for the used SWIR lens and the four different bandpass filters (910 nm, 980 nm, 1100 nm, and 1200 nm, FWHM of 10 nm each).

The more sophisticated filter integration layout is the so-called internal assembly (Section 2.1.3) and consists of a specially designed c-mount flange that mounts only one (hybrid) filter element between lens and sensor (see Figure 5) for spectral band selection. The transmission curves in Figure 19 show the transmission properties of the four different bandpass filters used. As described in Section 2.1.3, these consist of several different elements and thus achieve an ultra-wide blocking from 350 nm up to 2500 nm and therefore do not require any additional shortpass filter element. The measured transmissive properties are plotted in Figure 19 and show the advantage of these filters as well as confirm the manufacturer’s specifications. Over the entire sensitivity range of the InGaAs sensor, they have an almost perfect blocking down to the desired passband ranges of the bandpass filters. Besides, the filter element can easily be inserted into the optical path via a special flange.

### 4.3. Flat-Field Measurements

**Figure 20:**
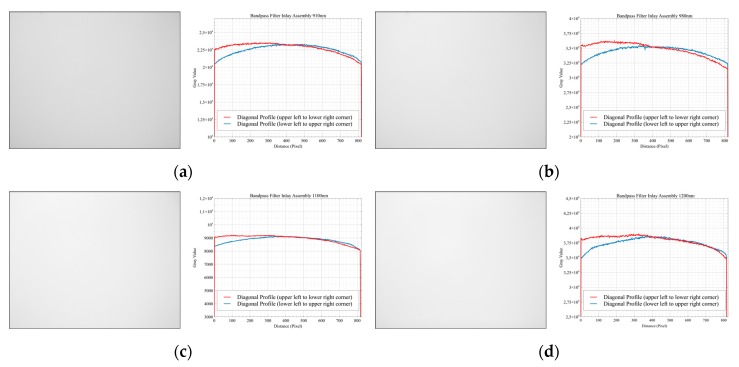
Flat-field image data from internal assembly layout with the corresponding diagonal image profiles plotted: (**a**) 910 nm; (**b**) 980 nm; (**c**) 1100 nm; (**d**) 1200 nm.

The optical characterization of the internal assembly approach was tested in a further step. For this purpose, the camera modules were equipped with the specially manufactured camera flanges (Section 2.1.3). The hybrid filter elements were inserted beforehand into the corresponding flanges. Figure 20 shows the results of the flat-field measurements on the integrating sphere. In contrast to the above-mentioned, discarded front-assembly solution, no artifacts are visible for all investigated wavelengths in the flat-field data. This is also confirmed by the vertical image profiles of the individual images. Only a drop in intensity towards the edges of the image, the so-called vignetting effect, can be seen, which was expected and can be corrected.

### 4.4. UAV Integration, System Test, and Test Flight of the Newly Developed VNIR/SWIR Imaging System

For the UAV integration, the SCU with the internal assembly approach of the filters was chosen. As described in Section 2.1.3, the artifacts occurring in the front assembly layout make this spectral filter solution unusable for use in the camera system. In contrast, the internal assembly layout (Section 2.1.3) does not generate any ambiguous artifacts (Section 4.3) and has therefore been selected to be integrated into the SCU for further use. The main features of this newly developed VNIR/SWIR multispectral imager, as shown in Figure 1, are summarized in Table 2.

**Figure 21:**
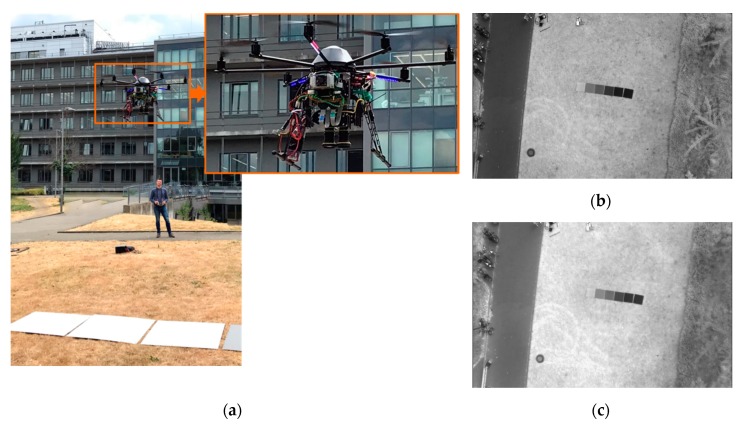
(**a**) The scenario for the test flight in which two image data sets with different wavelength bands were recorded; (**b**) Sample image of the acquired image data set from the 905 nm channel; (**c**) Sample image of the acquired image data set from the 1100 nm channel; The different reflection of the grass area between the wavelength and the nearly same reflection of the calibration panels and the concrete area is clearly visible.

**Figure 22:**
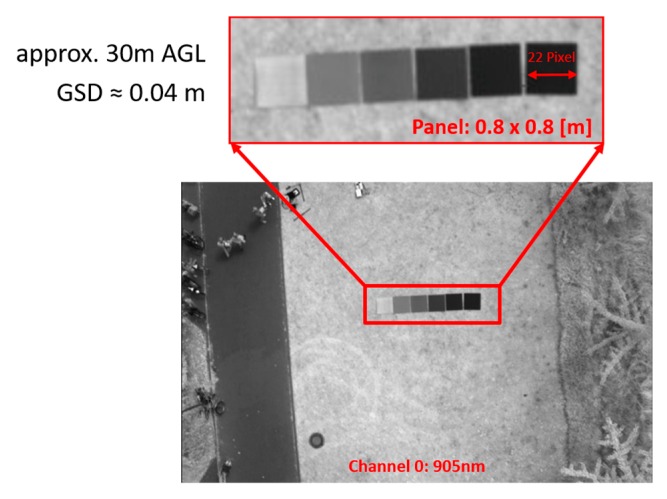
Calculations for the Ground Sampling Distance.

After the integration of the camera system into the carrier platform as described in Section 3.4 and successful tests in the laboratory, a first test flight at the outdoor area of the Institute of Geography (University of Cologne) was performed in the next step (Figure 21). Special attention was paid to possible shortcomings of the overall system during the flight. Particular attention was paid to the electromagnetic compatibility (EMC) of the individual components with regard to the high-current electronics of the rotor motors and to the influence of various vibration sources. Therefore, different flight situations, flight altitudes, and flight modes were tested. With the recorded test image data (Figure 22), the previously calculated ground resolution, the aperture settings on the lens as well as the optimal exposure time could be checked.

Based on the battery power used per time unit, a maximum flight time of around 14 min could be estimated for the whole system. Assuming a typical flight speed of 3 m/s, a ground altitude of 30 m, and an overlap of 75% across-track, an area of 2 ha could be easily covered, which is sufficient for a typical experimental field.

In order to ensure that the image data of the two cameras of the SCU cover the same area on the ground, the SfM software Metashape (Version 1.5.5, www.agisoft.com) is currently used in combination with precisely measured ground control points. Each image data set of a specific wavelength is processed individually, and the georeferenced orthomosaics can be further processed and evaluated in a geographic information system (GIS). For single image capture, a dedicated image-to-image registration is performed, and the previously distortion corrected images of a trigger event at a certain altitude are merged into a multi-layer tiff. This is currently done with MATLAB [63].

## 5. Discussion and Conclusions

Numerous studies indicate the higher potential for the VNIR/SWIR domain for remote sensing applications, e.g., vegetation trait estimations using wavelengths from the Red Edge to the SWIR domain [15,18,20,24,25,27,28,30,31,64,65,66,67,68,69]. While this spectral range is available for airborne- or satellite-borne pushbroom sensing systems, for UAV platforms, only a few first studies are known [14,15]. According to the authors, this is due to the fact that, in contrast to the VIS/NIR domain, there is currently no easy-to-use multispectral multi-camera system that also covers the SWIR spectral range.

There are different imaging systems with different sensor technologies that cover the VNIR and the SWIR spectral range for use with UAVs. Aasen et al. [13], as well as Hagen and Kudenov [61], provide a comprehensive review of the particular methods and systems. In the field of UAV-based SWIR imaging, especially pushbroom spectrometers (line scanners) [5,6,15,70,71] and partially sequential 2D imagers are used for data acquisition. Sequential 2D imagers use a tunable Fabry-Pérot Interferometer (FPI) to set the desired spectral band [7,72,73]. The advantages are the high spatial resolution and the flexible selection of the wavelength bands. However, the scanning time depends directly on the number of bands required, which also affects other parameters such as exposure time, flight altitude, and flight speed [13]. Pushbroom scanners offer a high spatial and spectral resolution, which, however, is only achieved in complex post-processing through the individual spectrally and locally resolved line data. In addition to the scanner itself, a powerful computer with a large memory capacity for the spectral data and additional modules for exact georeferencing (GNSS, IMU) have to be used.

In contrast to pushbroom spectrometers, multispectral multi-camera imagers offer the advantage of concise and lighter sensor structures, a high spatial resolution, and more straightforward data processing and evaluation methods, however, with the disadvantage of significantly reduced spectral information. They are therefore applied to use cases where the selection of spectral channels relevant to the problem has already taken place in previous studies and has been established, e.g., in vegetation indices to facilitate further investigation of a relevant topic.

Although there are many multi-camera imaging systems for the VNIR range [37,38,74,75,76], there is currently no multi-camera imaging system with a spectral response in the SWIR range (from 1000 nm upwards). Therefore, the first modular prototype of a multispectral multi-camera system using two VNIR enhanced SWIR imagers (approx. 400–1700 nm), and a powerful single-board computer was developed that is lightweight and compact enough to be carried by UAVs with a sensor payload capacity starting from 1.5 kg. Furthermore, the spectral image data of this newly develop VNIR/SWIR imaging system can be used for Structure from Motion (SfM) and Multi-View Stereopsis (MVS) analysis, resulting in spectral 3D data from one sensor [1,17]. Several authors already investigated the benefits of combined spectral and structural data analysis of vegetation [23,77]. As Roberts et al. [35] and Camino et al. [15] show, for monitoring vegetation traits, only a few VNIR/SWIR analyses require more than two to four spectral bands.

Two mounting devices, the so-called external and internal assembly, for placing the individual filters in the optical path of the respective sensor were successfully designed, built, and tested. The external filter positioning in front of the lens, realized by a snap-on adapter, is the more straightforward and, therefore, the most used setup for multispectral multi-camera systems. During imaging tests, however, this solution has proven to be unsuitable, as ring-like mirror artifacts have occurred. Therefore, this layout was discarded for further use at an early stage of development. The so-called internal assembly solution, however, positions the individual filter between lens and sensor via a specially developed and self-manufactured stainless steel C-mount flange. Each filter used is inserted into its own flange, which is specially adapted to the optical system. This means that the entire flange is always replaced when the filter is changed. In contrast to the rejected front-assembly layout with the developed snap-on filter adapter, this flange design has even more advantages.

On the one hand, the light beams emerging from the lens are more parallel, and a significant angle-dependent wavelength shift (blue-shift) [16] could not be detected in the spectroradiometer measurements. On the other hand, there are no ring-like mirroring artifacts generated by back reflections in the images, as the flat-field measurements have shown. In addition, the hybrid filters offer excellent blocking over the entire sensitivity range of the InGaAs sensor, so that only one filter element is required instead of two for the front-assembly approach. Due to the smaller diameter (25.4 mm), this solution is also less expensive than filter discs with a larger diameter (50 mm).

The spectral camera unit (SCU) was intensively tested for various parameters, both in a climatic chamber and with an integrating sphere. The tests in the climatic chamber included a preliminary characterization of the thermal noise behavior of the individual camera sensors and their internal electronics both at different temperatures and exposure times. The determined parameters of the Average Dark Current and the Fixed Pattern Noise provide information at the pixel level and are indispensable for further corrective measures since InGaAs sensors in particular exhibit higher temperature-induced noise values compared to silicon sensors. During the test flight it was shown that in daylight conditions exposure times between 1 ms, cloudless sky, and 4 to 6 ms, cloudy sky, the sensors are still sufficiently illuminated so that not the noise part of the sensor but the shot noise of the photons is the dominant part of the SNR. Furthermore, the effectiveness of the specially developed external fan system was tested.

With activated fans, a significant reduction and stabilization of the sensor temperature of 6 °C relative to the ambient temperature could be achieved. This is especially important in situations where the ambient temperature causes the internal temperature, especially the sensor temperature, to rise to a critical level. The influence of the fans on the cameras with regard to EMI or motion artifacts due to vibrations could not be detected in the image data.

The tests in the spectral laboratory included the validation of the filter characteristics of the two developed layouts (front- and internal assembly) as well as a preliminary characterization of the complete optical system (flat-field) with both filter assembly solutions. Only the internal assembly design proved to be suitable for further use since the front-assembly layout generated ring-like artifacts in the image data due to mirroring effects. The modular system was then successfully integrated into a multirotor UAV and tested in aerial operation.

The current state of development of the system allows the simultaneous image data recording of two wavelength bands. For collecting further bands, the equipped filter combinations have to be exchanged, and the examination area has to be flown over again. In stable weather conditions, this is no problem, because the image data of the newly developed prototype can easily be georeferenced via precisely measured ground control points. Additionally, spectral reference panels, as shown in Figure 21b,c and Figure 22, should be placed for each flight to ensure spectral ground-truth data. The UAV-based acquisition of overlapping image data with 0.04 m spatial resolution with two spectral bands takes for 1 ha approx. 7 min. In total, the data acquisition of four different VNIR/SWIR bands takes about 20 min (including filter change). Larger areas of up to 10 ha can be covered in lower resolution (approx. 0.13 m) at the same time. The latter resolution would be still precious to use calibrated VNIR/SWIR data from UAV data acquisition, also for validating satellite-borne remote sensing data.

Due to the remaining payload capacity of the UAV and the backend connectivity, up to two additional cameras can be added to the system to capture four spectral bands simultaneously. Although a quadruple VNIR/SWIR camera system would be the optimal solution, the first approach could be to cover the NIR range with two additional NIR-enhanced CMOS cameras based on silicon. These cameras are cheaper to acquire and even more compact and lighter than the OWL 640 Mini.

This study presents the novel development setup and initial validation of the first VNIR/SWIR imaging system for extending the possible spectral range to the SWIR domain in the field of UAV-based multi-camera systems. This new VNIR/SWIR imaging system for UAVs will enable innovative studies for remote sensing applications, e.g., to analyze non-destructively vegetation traits with a high spatio-temporal resolution. The next steps are the validation and calibration of the newly developed system for continuous vegetation monitoring for biomass, nitrogen, and stresses to verify its suitability for mapping VNIR/SWIR-based VIs such as NRI or GnyLi. Although the prototype already performs as expected and provides image data under real test conditions, it needs to be comprehensively tested and validated in remote sensing projects. Therefore, several UAV campaigns have been conducted for a grassland experiment, where spectral ground-truth data and vegetation traits were and will be collected simultaneously. In 2020, these activities will be extended to field experiments of crops, monitoring of silvopastoral systems, and forest applications.

## Figures and Tables

**Table 1:** Specifications of the applied hybrid bandpass filters (www.thorlabs.com).

CWL ^1^ (nm)	FWHM ^2^ (nm)	T ^3^ (%)	Blocking ^4^ (nm)	D ^5^ (mm)
910 ± 2	10 ± 2	≥50	200–2500	25.4
980 ± 2	10 ± 2	≥50	200–2500	25.4
1100 ± 2	10 ± 2	≥40	200–3000	25.4
1200 ± 2	10 ± 2	≥40	200–3000	25.4

^1^ Center Wavelength; ^2^ Full Width Half Max; ^3^ Peak Transmission; ^4^ < 0.01% (< −40 dB); ^5^ Diameter.

**Table 2:** Feature overview of the newly developed VNIR/SWIR imaging system.

Parameter	Specified Value
Sensors	InGaAs PIN-Photodiode
Data acquisition	Multi-Camera 2D imager
Spectral response	400 to 1700 nm
SNR_Peak_	58 dB
Dynamic Range	71 dB
Shutter mode	Global Shutter
Power Supply	9 to 36 V
Power consumption	15 W @ 12 V
Weight:	
SMU	600 g
SCU	900 g
